# Genetic polymorphisms are associated with serum levels of sex hormone binding globulin in postmenopausal women

**DOI:** 10.1186/1471-2350-9-112

**Published:** 2008-12-17

**Authors:** José A Riancho, Carmen Valero, María T Zarrabeitia, María T García-Unzueta, José A Amado, Jesús González-Macías

**Affiliations:** 1 Department of Internal Medicine, Hospital UM Valdecilla-IFIMAV, University of Cantabria, Santander, Spain; 2 Unit of Legal Medicine, University of Cantabria, Santander, Spain; 3 Laboratory of Endocrinology, Hospital UM Valdecilla, University of Cantabria, Santander, Spain

## Abstract

**Background:**

Estrogen activity plays a critical role in bone homeostasis. The serum levels of sex hormone binding globulin (SHBG) influence free estrogen levels and activity on target tissues. The objective of this study was to analyze the influence of common polymorphisms of the *SHBG *gene on serum SHBG, bone mineral density (BMD), and osteoporotic fractures.

**Methods:**

Four biallelic polymorphisms of the *SHBG *gene were studied by means of Taqman assays in 753 postmenopausal women. BMD was measured by DXA and serum SHBG was measured by ELISA.

**Results:**

Age, body weight, and two polymorphisms of the *SHBG *gene (rs6257 and rs1799941 [A/G]) were significantly associated with serum SHBG in unadjusted and age- and weight-adjusted models. Alleles at the rs1799941 locus showed the strongest association with serum SHBG (p = 0.0004). The difference in SHBG levels between women with AA and GG genotypes at the rs1799941 locus was 39%. There were no significant differences in BMD across SHBG genotypes. The genotypes showed similar frequency distributions in control women and women with vertebral or hip fractures.

**Conclusion:**

Some common genetic variants of the *SHBG *gene, and particularly an A/G polymorphism situated in the 5' region, influence serum SHBG levels. However, a significant association with BMD or osteoporotic fractures has not been demonstrated.

## Background

Osteoporosis is a complex disease characterized by reduced bone mineral density (BMD) and a propensity for fractures that results from the interaction of genetic and environmental factors. It has been estimated that the heritability of BMD is about 50–80% [1,2]. Estrogens play a critical role in bone homeostasis, and are essential for the acquisition and maintenance of bone mass. Estrogen deficiency accounts for the marked decrease in BMD when gonadal function ceases at the menopause, leading to the high incidence of osteoporotic fractures in postmenopausal women. However, it must be stressed that estrogens may still play a role after the menopause. In fact, the aromatization of androgenic precursors in the adipose tissue and other extragonadal tissues continues during life and an association between serum estradiol and certain polymorphisms of the aromatase gene has been reported [3]. Similar to other lipophilic hormones, estradiol circulates in the blood bound to proteins, specifically to sex hormone binding globulin (SHBG). The free fraction of the hormone is the fraction usually considered to be the biologically important form. As a consequence, the levels of SHBG may influence estradiol bioavailability and cellular effects. Thus, it is important to understand the factors that modulate SHBG levels. Some of the factors are well-known, including pregnancy, exogenous sex steroids, and body weight [4]. In recent years, some studies have suggested that certain genetic variants of the *SHBG *gene could also influence SHBG levels and estrogen activity on both normal and neoplastic target tissues. However, the available data are limited and the reported results are controversial [5-13]. Therefore, the objective of this study was to analyze the influence of several polymorphisms of the *SHBG *gene on SHBG serum levels, BMD, and osteoporotic fractures

## Methods

### Individuals

We studied a group of 753 postmenopausal women over 60 years of age. They were volunteers recruited by voice and written announcements or sent to our outpatient clinic to be studied for possible osteoporosis (n = 463; age, 70 ± 6 yr), and women admitted to the hospital with hip fractures due to low-energy trauma (n = 290; age, 78 ± 6 yr). All participants were living in Cantabria, a region in Northern Spain with a population of about 530,000. They were interviewed by one of the investigators in order to check the absence of exclusion criteria. Subjects taking bisphosphonates, corticosteroids, antiepileptics, estrogens, or other drugs known to modify bone mass, or with non-Spanish ancestry, were excluded. The study was approved by the Institutional Committee on Ethics in Clinical Research.

### Techniques

BMD was determined in control women and women with spine osteoporosis by anterior-posterior DXA scans at the lumbar spine (L2–L4) and the total hip region using an Hologic QDR 4500 densitometer (Hologic, Waltham, MA, USA). Serum SHBG was measured by ELISA (IBL, Hamburg, Germany) with a sensitivity of 0.2 nmol/l and a coefficient of variation of 9%.

### Genetic analysis

We used PupaSuite (available at ) and FastSNP/VisualSNP (available at ) to identify potentially functional single nucleotide polymorphisms in the *SHBG *gene. Those web-based tools utilize several algorithms to identify sequences likely to influence gene transcription or the function of the gene product based on a number of features, such as the homology to transcription factor binding sites, the coincidence with splicing sites, or amino acid changes [14-16]. We chose variations with a minor allelic frequency > 5% in Caucasian populations and thus selected rs6260, rs6257, and rs6259. We also included rs1799941 based on some literature reports suggesting an association with SHBG levels [8]. Genomic DNA was obtained from the peripheral blood using a commercial kit, according to the manufacturer's instructions (Qiagen, Hilden, Germany). SHBG genotyping was performed by means of a procedure based on the exonuclease activity of Taq DNA-polymerase, using allele-specific Taqman probes labelled with VIC and FAM. Primers and probes were designed by the manufacturer with Primer Express software (Applied Biosystems, Foster City, CA, USA). After amplification in an ABI9700 thermal cycler (Applied Biosystems), the fluorescence was read in an ABI7300 sequence detector. Random samples were analysed twice to check for consistency of results.

### Statistics

Hardy-Weinberg equilibrium (HWE) was checked with HWSIM software (available at ). Linkage disequilibrium measures were estimated with Haploview software [17]. SHBG values did not follow a normal distribution, thus their natural logarithms were computed prior to statistical analysis. Between-genotype comparisons were done by means of general linear models, with and without adjustment for possible confounders, and by linear regression, assuming an additive genetic model by coding genotypes numerically as 1,2 (heterozygotes) and 3, and using SPSS software (SPSS Inc., Chicago, IL, USA). Power calculations were done with Quanto software (Gauderman WJ, Morrison JM. QUANTO 1.2: A computer program for power and sample size calculations for genetic-epidemiology studies, , 2006).

## Results

The rs6260 locus had very little variation in our population, with a minor allele frequency of 0.5% and was thus not considered further in the analysis. The minor allelic frequencies for rs6257 (C), rs6259 (A), and rs1799941 (A) were 16%, 15%, and 27%, respectively. There was no evidence for deviation from the HWE. The three polymorphisms exhibited significant linkage disequilibrium (rs1799941-rs6259: D' = 1.0, r^2 ^= 0.05, p < 0.001; rs1799941-rs6257: D' = 0.81, r^2 ^= 0.03, p = 0.03; and rs6257-rs6259: D' = 1.0, r^2 ^= 0.01, p = 0.01).

**Figure 1:**
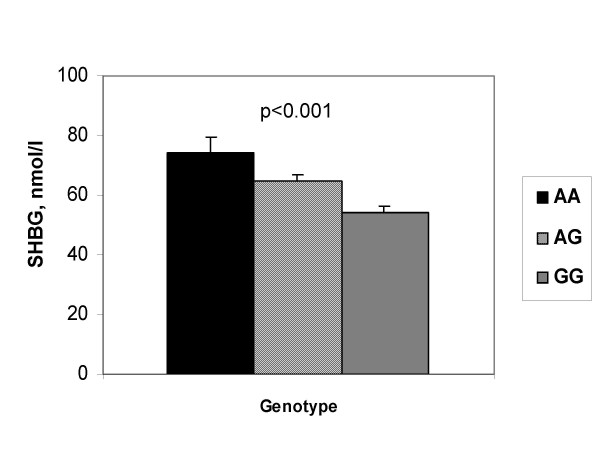
**SHBG levels according to rs1799941 (mean and SEM of age- and weight-adjusted values)**.

Based on univariate regression analysis, age, body weight, and the polymorphisms were significantly associated with serum SHBG levels measured in 307 randomly selected women of the control and osteoporotic subgroups. Among the polymorphisms, rs1799941 and rs6257 showed a stronger association with serum SHBG than rs6259 (Table 1). When the results were adjusted by age and weight, the associations of rs1799941 and rs6257 genotypes with serum SHBG remained statistically significant (p = 0.0004 and 0.009, respectively). In Figure 1, the age and weight-adjusted SHBG levels across rs1799941 genotypes are shown. The SHBG levels in AA homozygotes were 39% higher than in GG homozygotes. Based on multiple linear regression models, age and weight together accounted for 10.5% of the variance of serum SHBG; the inclusion of the rs1799941 genotype explained a further 3.7% of the SHBG variance. The inclusion of other polymorphisms did not further increase the proportion of variance explained.

**Table 1:** Factors associated with serum SHBG levels (Beta standarized regression coefficients and p values from univariate and multivariate regression analyses)

	Univariate		Age- and weight-adjusted	
	Beta	p	Beta	p

Age	0.17	0.002		
Weight	0.28	0.00006		
rs6257	0.15	0.010	0.14	0.009
rs1799941	0.14	0.0015	0.19	0.0004
rs6259	0.11	0.048	0.06	0.27

Serum SHBG showed a negative association with BMD (p = 0.040 at the spine and 0.014 at the hip). Thus, we next determined the association between the rs1799941 allele and BMD and osteoporotic fractures; however, there were no genotype-related significant differences in the spine or hip BMD (Table 2), nor in the distribution of genotypes in women with and without fractures (Table 3). The study had 87% power to detect an association between rs1799941 polymorphisms and BMD if at least 2% of the BMD variance was explained. Likewise, the power to detect an association with vertebral and hip fractures was 87% and 96%, respectively, assuming a risk ratio of 1.6.

**Table 2:** Spine and hip BMD according to rs1799941 genotypes (mean (SE) and 95% confidence intervals after age- and weight-adjustment)

	Spine			Hip		
	
Genotype	Mean (SE)	95% CI	p	Mean (SE)	95% CI	p
AA	0.815 (0.027)	0.763–0.868	0.9	0.807 (0.021)	0.766–0.848	0.9
AG	0.820 (0.013)	0.794–0.845		0.799 (0.010)	0.779–0.819	
GG	0.823 (0.010)	0.803–0.843		0.802 (0.008)	0.786–0.818	

**Table 3:** Distributions of rs1799941 genotypes in control women and women with osteoporotic fractures (numbers (%) and odds ratios considering the most frequent genotype as the reference)

	Controls	Vertebral fractures		Hip fractures	
Genotype	n (%)	n (%)	OR (95% CI)	n (%)	OR (95% CI)

AA	24 (7)	9 (6)	1.0 (0.5–2.3)	19 (6)	0.8 (0.4–1.5)
AG	125 (40)	54 (37)	1.2 (0.8–1.8)	107 (37)	0.9 (0.6–1.2)
GG	169 (53)	62 (57)	reference	164 (57)	reference

Total	318 (100)	145 (100)		290 (100)	

## Discussion

The *SHBG *gene is located on chromosome 17 and the major transcript is encoded by 8 exons, spanning approximately 3.2 Kb. In the present study, we report that several polymorphisms of the *SHBG *gene are strongly associated with serum SHBG levels in postmenopausal women. In particular, women with the AA genotype at the rs1799941 locus had 39% higher SHBG levels than those with the GG genotype. This polymorphism is located in the 5' region of the gene, 8 nucleotides upstream of the transcription start site. Thus, it is well situated to influence gene transcription. In fact, higher SHBG serum levels have also been recently reported in men and women bearing A alleles, in comparison with those with G alleles. Dunning et al. [18] found that SHBG serum levels were 28% higher in women with the AA genotype than in those with the GG genotype. Similarly, Eriksson et al. [8] found 22% and 26% higher SHBG levels in individuals with the AA genotype in two cohorts of Swedish men. The C/T rs6257 polymorphism was also associated with SHBG levels in the present study. The mechanism is unclear, as it is located in an intronic region (intron 1). A search of potentially functional polymorphisms with the PupaSuite web-based tool revealed that it is located in a potential binding site for the transcription factor hepatocyte nuclear factor 3/Fox. The A/G rs6259 polymorphism is located in exon 8 and causes a change in the amino acid sequence of SHBG (Asn>Asp). It was also associated with SHBG levels, but to a lesser extent than the other polymorphisms. It has been suggested that the Asn>Asp modification affects a potential glycosylation site and increases the half-life of the protein coded for the less common allele [6]. Previous studies of the relationship of this SNP with serum SHBG in women have given conflicting results [6,9,18].

Our results show a clear association between several *SHBG *gene polymorphisms and serum SHBG levels, but since they are in linkage disequilibrium, we cannot establish with certainty which polymorphism is truly responsible for the association. On the other hand, we cannot exclude the possibility that other polymorphisms in linkage disequilibrium with those studied herein are actually responsible for the association with SHBG levels [6,8]. Serum SHBG showed a stronger association with rs1799941 than with other loci. However, allelic frequencies of rs1799941 were more balanced than those of other polymorphisms, which may favour the detection of associations between genotypes and SHBG levels.

Several authors have reported an association between serum SHBG and BMD or osteoporotic fractures [19-24]. In the present study, we did not find an association between *SHBG *alleles and BMD, despite the fact that they were strongly associated with serum SHBG levels, which in turn was associated with BMD. This lack of association between *SHBG *genotype and BMD may be due merely to the multiple factors influencing these traits and introducing noise in the relationship between genotype, serum SHBG levels, and BMD. Although the association between genotypes and serum SHBG was statistically significant and quantitatively important (39% difference between AA and GG genotypes), there was wide inter-individual variation, and thus rs1799941 alleles explained < 4% of serum SHBG variance. Similarly, the contribution of serum SHBG to BMD variance was rather small (SHBG explained 2% of the BMD variance at the spine and 3% at the hip). Thus, given the moderate sample size of this study, we cannot completely exclude a small effect of *SHBG *polymorphisms on BMD or fractures, but our data suggest that such effect, if any, is unlikely to be clinically important in postmenopausal women.

It is also worth noting that SHBG may have a complex influence on estrogen activity and, consequently in the homeostasis of estrogen-responsive tissues, such as bone. It is generally accepted that the unbound fraction of sex steroids is the biologically important fraction because it can diffuse from the capillaries into the peripheral tissues. However, SHBG may be synthesized in certain target tissues and some cells appear to have specific binding sites for SHBG and actively internalize it, through the megalin and other pathways [25-27]. Although the true biological significance of this phenomenon is unclear, it might represent a mechanism increasing estrogen activity on the target tissues. The results of some studies appear to be in line with this hypothesis. In fact, although a negative association between SHBG and BMD or fractures was reported in several studies [19-21] others found a direct positive correlation between serum SHBG levels and hip BMD [8]. Thus, it has been suggested that increasing SHBG may either facilitate or impair estradiol action on target tissues, depending on several factors, including the overall estrogen status [25].

The results of genetic association studies may be biased by a number of factors, including genotyping and phenotyping errors, and population stratification. Good clinical and laboratory practices can diminish the impact of such errors, but the confounding effect of population substructure may be particularly difficult to exclude [28]. Nevertheless, we tried to avoid it by studying women in a relatively small region and excluding those with non-Spanish ancestors. Given the moderate size of our study, a relatively small, but still clinically significant effect (i.e., a 1.2 odds ratio for fracture) cannot be excluded. On the other hand, we cannot exclude the possibility that other polymorphisms in the *SHBG *gene region might have an influence on BMD or fracture risk. The *SHBG *gene is small and only a few SNPs are included in the Hapmap database. Therefore, we focused on potentially functional SNPs, rather than in haplotype-tagging SNPs.

## Conclusion

Our results show that apart from other factors, such as age and body weight, some common genetic variants of the *SHBG *gene, and particularly an A/G polymorphism situated in the 5' region, influence serum SHBG, but we have not been able to demonstrate its association with either BMD or osteoporotic fractures.

## Competing interests

The authors declare that they have no competing interests.

## Authors' contributions

JAR conceived and coordinated the study, carried out the statistical analysis and wrote the manuscript draft. CV was responsible for the bone densitometry studies. MTZ was responsible for the genetic studies. MTGU carried out the biochemical analyses. JAA participated in the design of study and supervised the biochemical analyses. JGM helped in the design of the study and made substantial contributions to the interpretation of results. All authors reviewed the first draft and read and approved the final manuscript.

## Pre-publication history

The pre-publication history for this paper can be accessed here:


